# Cobalt oxide nanoparticles induce oxidative stress and alter electromechanical function in rat ventricular myocytes

**DOI:** 10.1186/s12989-020-00396-6

**Published:** 2021-01-06

**Authors:** Monia Savi, Leonardo Bocchi, Francesca Cacciani, Rocchina Vilella, Annamaria Buschini, Alessio Perotti, Serena Galati, Serena Montalbano, Silvana Pinelli, Caterina Frati, Emilia Corradini, Federico Quaini, Roberta Ruotolo, Donatella Stilli, Massimiliano Zaniboni

**Affiliations:** 1 Department of Chemistry, Life Sciences and Environmental Sustainability, University of Parma, Parco Area delle Scienze 11/a, 43124 Parma, Italy; 2 Centre for Molecular and Translational Oncology (COMT), University of Parma, Parma, Italy; 3 Department of Medicine and Surgery, University of Parma, Parma, Italy

**Keywords:** Cobalt oxide nanoparticles, Nanotoxicology, Cardiac EC coupling, Intracellular calcium dynamics, Genotoxicity, ROS production

## Abstract

**Background:**

Nanotoxicology is an increasingly relevant field and sound paradigms on how inhaled nanoparticles (NPs) interact with organs at the cellular level, causing harmful conditions, have yet to be established. This is particularly true in the case of the cardiovascular system, where experimental and clinical evidence shows morphological and functional damage associated with NP exposure. Giving the increasing interest on cobalt oxide (Co_3_O_4_) NPs applications in industrial and bio-medical fields, a detailed knowledge of the involved toxicological effects is required, in view of assessing health risk for subjects/workers daily exposed to nanomaterials. Specifically, it is of interest to evaluate whether NPs enter cardiac cells and interact with cell function. We addressed this issue by investigating the effect of acute exposure to Co_3_O_4_-NPs on excitation-contraction coupling in freshly isolated rat ventricular myocytes.

**Results:**

Patch clamp analysis showed instability of resting membrane potential, decrease in membrane electrical capacitance, and dose-dependent decrease in action potential duration in cardiomyocytes acutely exposed to Co_3_O_4_-NPs. Motion detection and intracellular calcium fluorescence highlighted a parallel impairment of cell contractility in comparison with controls. Specifically, NP-treated cardiomyocytes exhibited a dose-dependent decrease in the fraction of shortening and in the maximal rate of shortening and re-lengthening, as well as a less efficient cytosolic calcium clearing and an increased tendency to develop spontaneous twitches. In addition, treatment with Co_3_O_4_-NPs strongly increased ROS accumulation and induced nuclear DNA damage in a dose dependent manner. Finally, transmission electron microscopy analysis demonstrated that acute exposure did lead to cellular internalization of NPs.

**Conclusions:**

Taken together, our observations indicate that Co_3_O_4_-NPs alter cardiomyocyte electromechanical efficiency and intracellular calcium handling, and induce ROS production resulting in oxidative stress that can be related to DNA damage and adverse effects on cardiomyocyte functionality.

**Supplementary Information:**

The online version contains supplementary material available at 10.1186/s12989-020-00396-6.

## Background

In the last decades, technology, bioengineering and medicine have focused on the use of nanomaterials, due to their unique chemical, physical, and biological properties [1–3], and new possible fields of application are extensively explored. The massive production of nanoparticles (NPs) that can be delivered into the environment represents though a source of risk, particularly for workers involved in NP manufacturing, who are exposed to high NP concentrations for many hours a day. A precise estimate of individual exposure has proved to be difficult [4]. Nanotoxicology is aimed at characterizing the adverse effects of NPs on human health and understanding the molecular and cellular basis of these effects [5–7]. Physicochemical properties of nanosized materials like particle size, coating, morphology, and the capacity to form agglomerates may play a role in determining potential harmful effects on human health [8, 9].

We focused the present study on cobalt oxide (Co_3_O_4_) NPs (Co_3_O_4_-NPs), whose use is gaining increasing interest in many fields, due to their specific properties. For example, they have been proposed as a suitable negative contrast agent in MRI [10], as pigment, catalysts [11], and as a substitute for lithium in energy storage devices, due to NP supercapacitor properties [12] and to their improved cycling performance [13]. Cobalt is also utilized as a component of high-performance and wear resistant alloys that are critical in the manufacture of implanted medical devices (e.g. coronary artery stents and metal orthopedic prostheses) [14–16]. On the other hand, since 1960 the correlation between cobalt exposure and cardiomyopathy in humans has been a subject of debate [17–21]. In those years, an epidemic of cardiomyopathy occurred in Canada, the United States, and Belgium among people who drank large amounts of beer containing cobalt used to increase foam stability [17–21]. This cardiomyopathy was associated with a high rate of mortality (10–40%), severe biventricular heart failure, pericardial effusion, hypotension, and ECG abnormalities, in the absence of cardiac arrhythmias [17, 18, 21–23]. Post-mortem analyses revealed left ventricular (LV) chamber dilation and wall thickening. It was assumed that beer-drinker cardiomyopathy was a multifactorial disease which developed when short-term exposure to cobalt was associated with other factors like low-protein diet, thiamine deficiency, alcoholism, and hypothyroidism [20, 21].

More recently studies on cardiac function in cobalt-exposed workers have revealed no impairment of LV systolic function [24, 25]. However, some reports described heart failure and cardiogenic shock [26, 27], or the occurrence of hypertension and reversible ECG changes (depressed ST and T waves, arrhythmias) [28, 29], in individuals exposed to cobalt. In 2004, Linna et al. [30] observed that prolonged exposure to cobalt was associated with altered LV diastolic function without signs of systolic cardiac dysfunction. Moreover, medical literature revealed 15 patients with cardiac disorders that were attributed to cobalt alloy prosthesis [31].

Most of the in vitro and in vivo experimental studies attributed cobalt or cobalt NP toxicity to an enhanced production of reactive oxygen species (ROS), alterations in DNA repair mechanisms, induction of DNA fragmentation, and mitochondrial dysfunction [32–41]. Specifically, ROS overproduction in the heart results in a depressed cardiac function and changes in cell structure [42–45].

In addition, Cho et al. [46] showed a massive pulmonary inflammatory response in rats instilled with Co_3_O_4_-NP_S_. It’s now well known that NPs can translocate from lungs to several body districts within minutes, depending on their size [47], and can trigger arrhythmias when reaching the heart [48, 49]. In light of all these findings and the implications for the risk management of occupational NP exposure, the National Institute of Occupational Safety and Health (NIOSH) recommends an exposure limit of 0.05 mg/m^3^ for Cobalt metal dust and fume, while the American Conference of Governmental Industrial Hygienists (ACGIH) fixed a limit of 0.02 mg/m^3^ for cobalt metal and inorganic compounds.

Recommended limits for exposure to cobalt metal and ionic cobalt, though, can hardly be extrapolated to cobalt NPs, and this makes the dosage for in vivo and in vitro experimental studies still largely arbitrary.

Taken together, these results highlight a potential negative correlation between cobalt NP exposure and cardiac function, as well as the need for both more comprehensive epidemiological reports and experimental mechanistic studies. To the best of our knowledge, although NPs have been shown to be able to translocate from lungs to the heart, the direct effects of Co_3_O_4_-NPs on cardiomyocyte function has never been investigated. Our working hypothesis is that direct interaction between NPs and cellular compartments might explain some of the harmful outcomes documented in clinical studies.

We specifically addressed this issue by analyzing the acute effects of Co_3_O_4_-NP exposure on electromechanical properties, ROS accumulation, and DNA damage of isolated ventricular myocytes. Transmission electron microscopy (TEM) was also used to detect the presence of Co_3_O_4_-NPs within cardiomyocytes.

## Results

### Cellular electrophysiology: resting potential and membrane capacitance

**Fig. 1:**
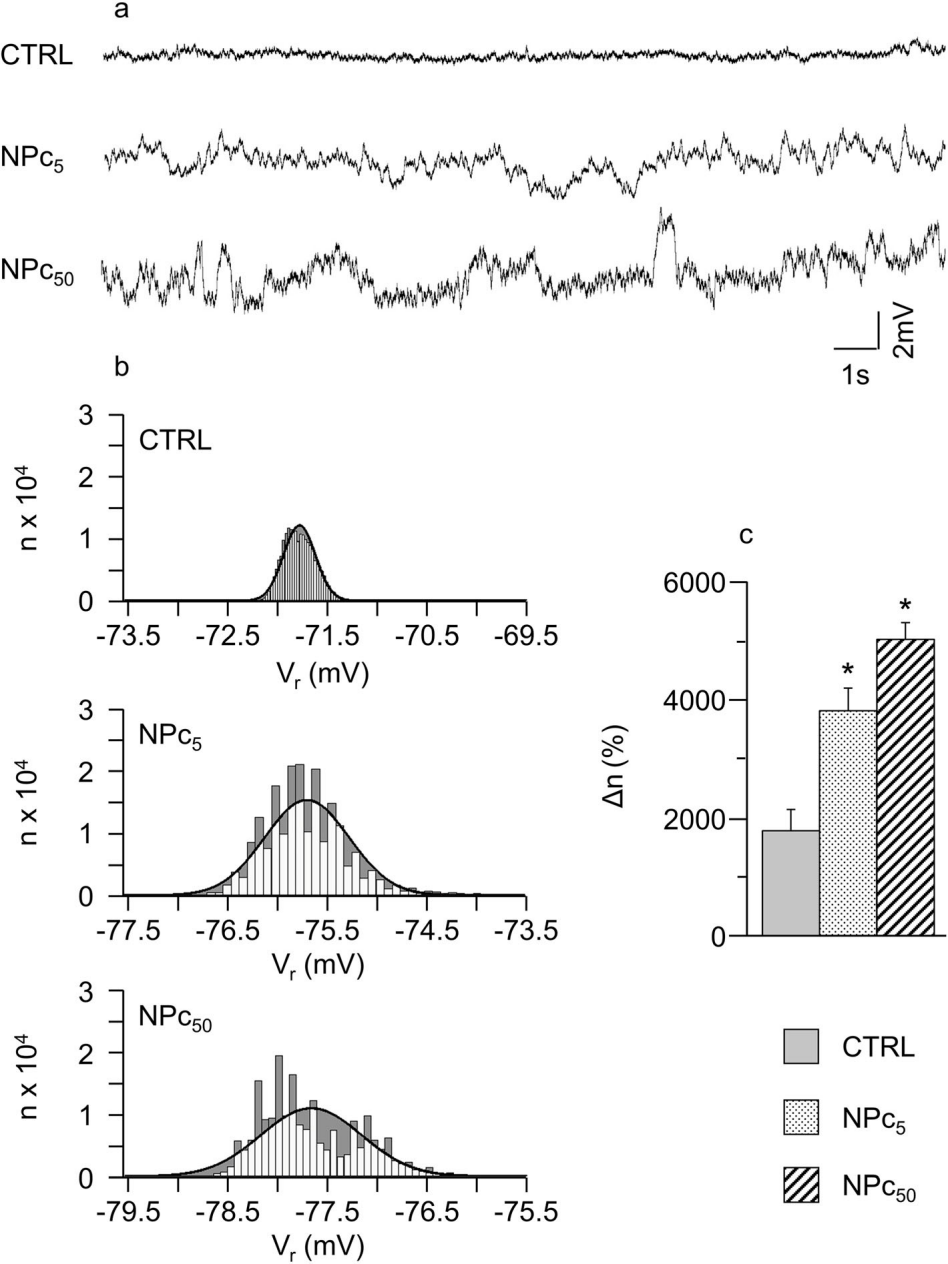
Variability of resting membrane potential following exposure to Co_3_O_4_-NPs. **a**) Representative 20 s V_r_ time courses recorded in un-stimulated patch clamped cells belonging to the three experimental groups, previously conditionally paced at 5 Hz for 8 s; **b**) frequency distributions of V_r_ (50 sub-intervals of the entire V_r_ range) with superimposed normal fitting (solid lines). The difference between the number of samples for each interval and that expected from the normal fitting (Δn) is marked in grey; **c**) mean values ± SEM of Δn, in CTRL, NPc_5_, and NPc_50_ groups. *, *p*< 0.05 vs CTRL (Kruskal-Wallis non-parametric statistical test followed by U-Mann Whitney test)

In order to investigate how the exposure to Co_3_O_4_-NPs affects resting membrane potential (V_r_), V_r_ variability of patch-clamped cardiomyocytes (*n*= 18 control cells, CTRL; 37 NP treated cardiomyocytes at 5 μg/ml, NPc_5_; 29 NP treated cardiomyocytes at 50 μg/ml, NPc_50_) was analyzed, in terms of statistical displacement from a normal distribution, in 20 s-long transmembrane potential (V_m_) recordings. It should be noted that none of the examined cells fired spontaneous action potentials (Aps) during the recording intervals. In Fig. 1a-b, representative examples of these recordings are reported for the three conditions under study.

Mean values and standard deviation (SD) of 20 s-long V_r_ recordings for each condition were used for Gaussian-fittings of the sampled data (solid curves in Fig. 1b). The difference (grey in Fig. 1b) between frequency distribution of the same data (ranked in 50 sub-intervals over the entire V_r_ range) and their Gaussian fit measures the number of beats responsible for deviation from normality, which is reported, normalized to the total number of beats of each sequence (Δn), in panel c of the same figure. Δn significantly increases in cells exposed to Co_3_O_4_-NPs at both doses.

Interestingly, in a number of cases, the distribution of V_r_ showed a characteristic two-band behavior like that reported in the lower panel of Fig. 1b, with a negligible incidence in CTRL (~ 15%), and a dose-dependent increase in NP-treated cells (up to ~ 41% in NPc_50_).

**Fig. 2:**
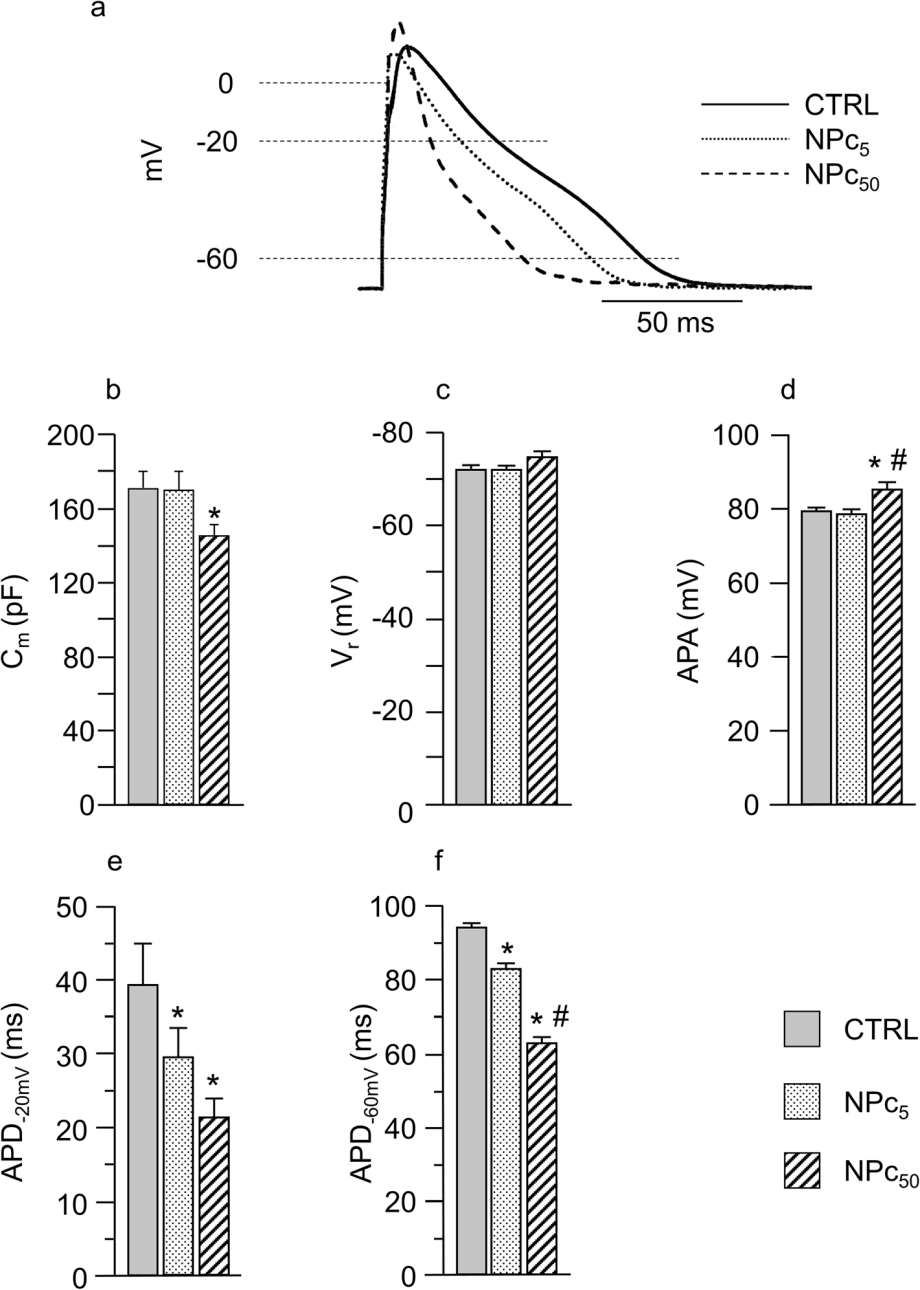
Effects of Co_3_O_4_-NPs on rat ventricular action potential. **a**) Representative superimposed AP waveforms recorded at the pacing rate of 5 Hz from the three cell groups. Panels **b**-**f**: mean values ± SEM of membrane capacitance (b, C_m_), resting membrane potential (c, V_r_), AP amplitude (d, APA), and AP duration at − 20 (e, APD_-20mV_) and − 60 mV (f, APD_-60mV_), recorded in CTRL (*n* = 21), NPc_5_ (*n* = 14), and NPc_50_ (*n* = 23) cardiomyocytes. *, p< 0.05 vs CTRL; #, p< 0.05 vs NPc_5_ (GLM-ANOVA for repeated measurements)

Membrane capacitance (C_m_) was also measured in 25 CTRL, 19 NPc_5_, and 23 NPc_50_ cardiomyocytes as an estimate of cell size, and was found significantly decreased in NPc_50_ in comparison with both, CTRL and NPc_5_ cells (Fig. 2b).

### Cellular electrophysiology: action potential (AP) parameters

AP measurements were performed on AP trains recorded as described in Methods. Figure 2a shows AP waveforms representative of the 3 experimental groups, recorded at 5 Hz, which is approximately the rat physiological beating rate. AP parameters were measured in 21 CTRL, 14 NPc_5_ and 23 NPc_50_, and corresponding statistics are reported in Fig. 2c-f. No differences among groups were observed for the resting membrane potential (V_r_ range: − 70/− 74 mV; Fig. 2c) while the AP amplitude (APA) was significantly increased in NP_C50_ cardiomyocytes (approximately + 7%; Fig. 2d).

The most relevant electrophysiological alteration induced by acute exposure to NPs concerned AP profile. A dose-dependent reduction of action potential duration (APD) was found with respect to controls in the early phase of repolarization (APD_-20mV_) and, more markedly, in the late phase (APD_-60mV;_ *p*< 0.05). Specifically, NPc_5_ displayed a 25% shortening in APD_-20mV_ and a 12% in APD_-60mV_, while in NPc_50_ the percentage decrease in the same parameters was 46 and 34%, respectively (Fig. 2a, e-f). Beat-to-beat variability of APD_-60mV_, measured as coefficient of variability (CV_-60mV_), was not significantly altered following exposure to NPs (data not shown).

### Cell contractility and calcium dynamics

**Fig. 3:**
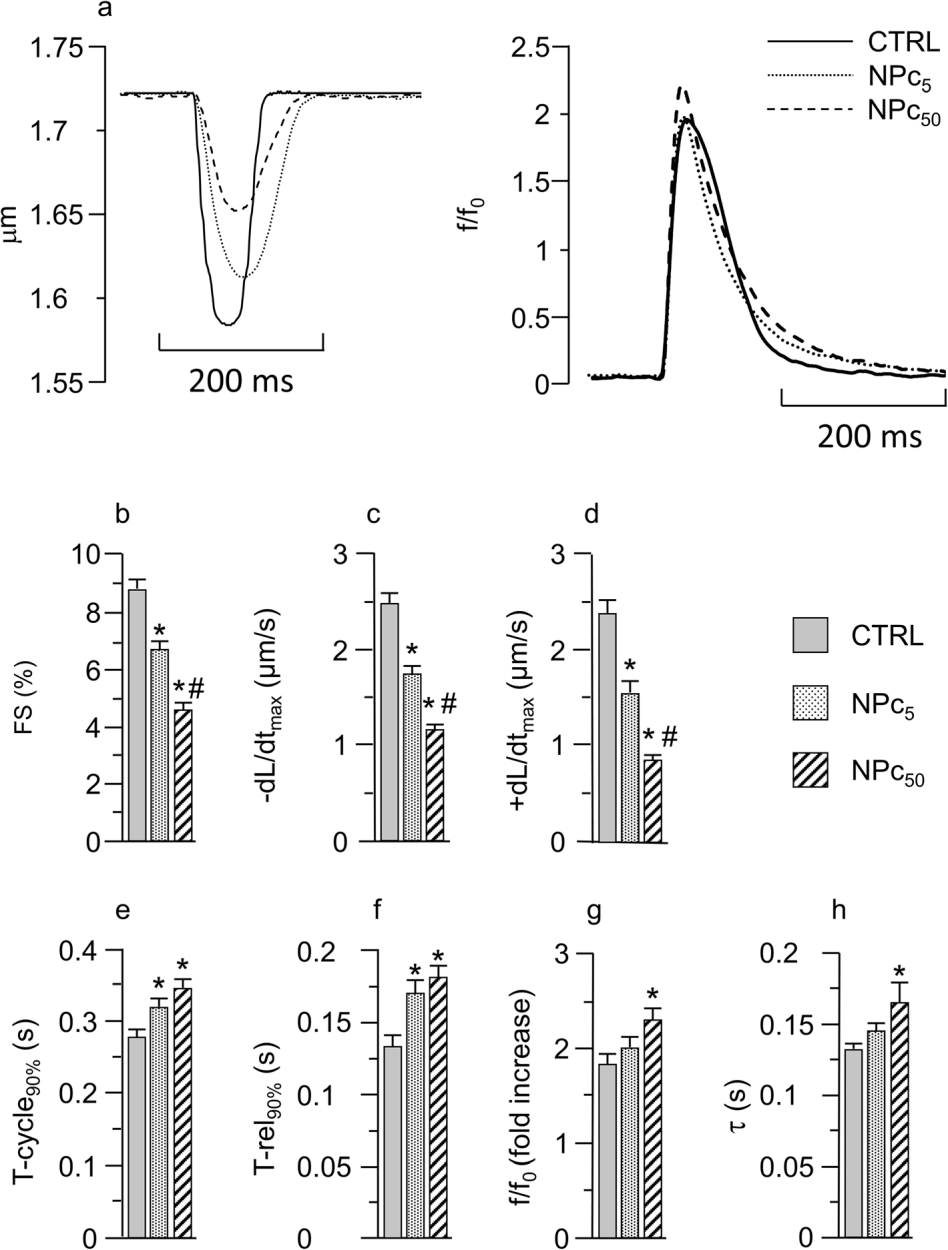
Effects of Co_3_O_4_-NPs on cardiomyocytes mechanics and intracellular calcium dynamics. **a**) Representative traces of sarcomere shortening (left panel) and calcium transients (right panel) recorded in CTRL, NPc_5_, and NPc_50_ groups. Panels **b**-**h**: mean values ± SEM of sarcomere shortening (b, FS), maximal rate of shortening (c, −dL/dt_max_) and re-lenghtening (d, +dL/dt_max_), time of total cycle measured at 90% of re-lengthening (e, T-cycle_90%_), and re-lengthening time measured from the peak of contraction (f, T-rel_90%_), calcium transient amplitude, expressed as fluorescence peak normalized to baseline (g, f/f_0_), and time constant of intracellular Ca^2+^ decay (h, τ) measured in CTRL (73 and 40 cells, for mechanics and calcium transients respectively), NPc_5_ (71 and 35), and NPc_50_ (88 and 21) cells. *, p< 0.05 vs CTRL; #, p< 0.05 vs NPc_5_ (GLM-ANOVA for repeated measurements)

Cellular mechanical properties were investigated in 73 CTRL, 71 NPc_5_, and 88 NPc_50_ cardiomyocytes. Representative recordings of sarcomere shortening (left) and Ca^2+^ transients (right) in the three groups of cells stimulated at 0.5 Hz are reported in Fig. 3a. The average diastolic sarcomere length was similar in all groups (1.73 ± 0.05 μm in CTRL, 1.71 ± 0.06 in NPc_5_, and 1.72 ± 0.04 in NPc_50_ cells), whereas contraction/relaxation properties and intracellular calcium dynamics were impaired in NP-treated cells. Specifically, the fraction of shortening (FS, %) and the maximum rate of shortening (−dL/dt_max_, μm/s) and relengthening (+dL/dt_max_, μm/s) exhibited a significant dose-dependent decrease in NP-treated cardiomyocytes in comparison with CTRL (Fig. 3b-d). Consistently, the duration of the entire cycle (T-cycle_90%_) and the re-lengthening phase (T-rel_90%_) (Fig. 3e-f) were prolonged to a comparable extent by both NP doses. The analysis of calcium dynamics, performed on 40 CTRL, 35 NP_C5_, and 21 NP_C50_ cells, has shown that the amplitude of Ca^2+^ transient (f/f0, fold increase; Fig. 3a, g) was increased by approximately 10 and 25%, in NPc_5_ and NPc_50_ respectively. A similar percent increment was observed for the time required for cytosolic calcium removal (τ; Fig. 3h). Statistically significant differences compared with CTRL were found only for the higher dose (Fig. 3g-h). Similar results were obtained when cardiomyocytes were stimulated at 1 Hz pacing frequency (data not shown).

**Fig. 4:**
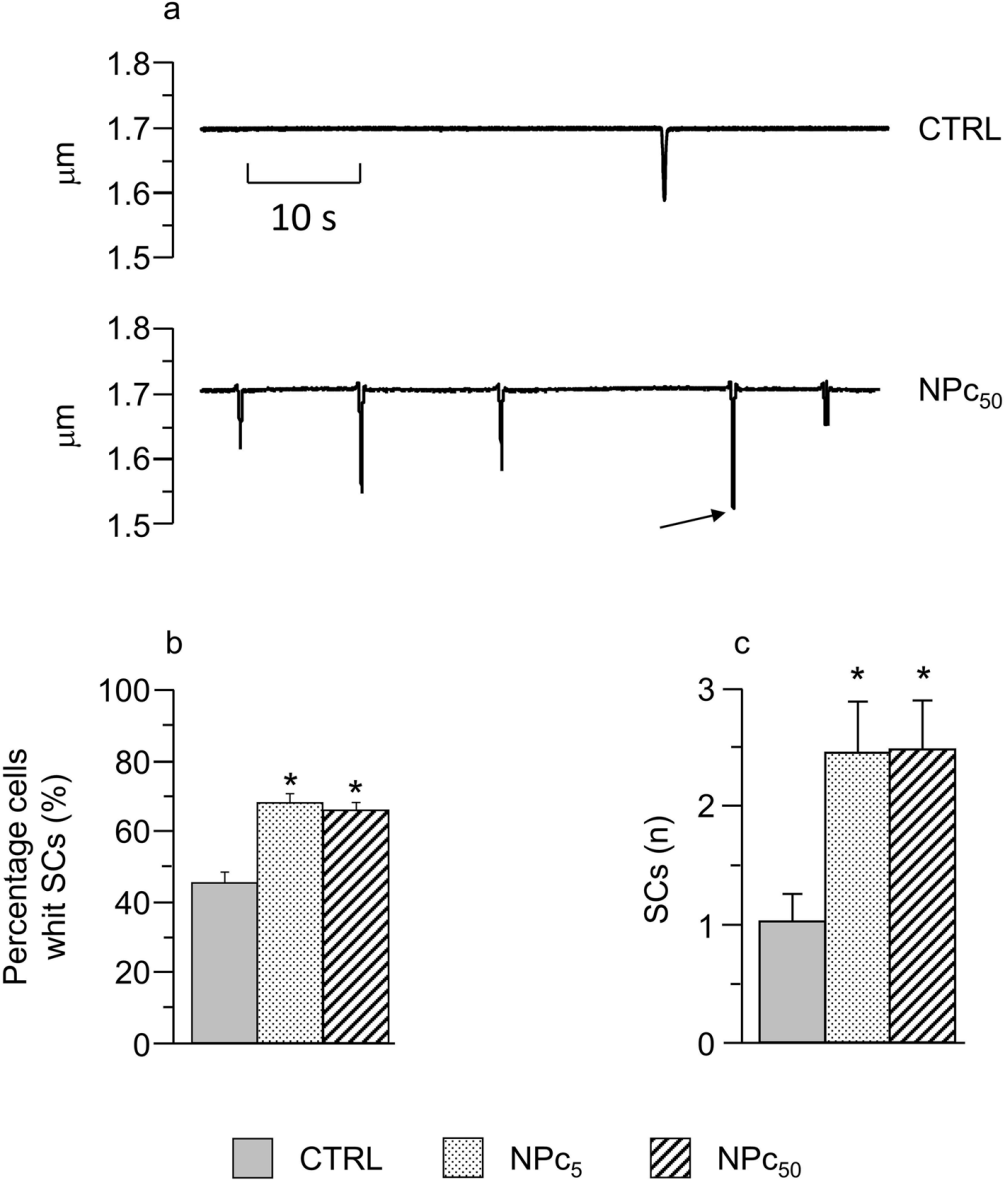
Effects of Co_3_O_4_-NPs on spontaneous cell contractions. **a**) Representative traces of spontaneous contractions (SCs) occurring in CTRL (top) and NPc_50_ (bottom) cardiomyocytes during 60 s recordings, after a 60 s conditioning pacing at 2 Hz. The arrow indicates a SC whose amplitude is comparable to an electrically induced twitch; **b**) mean values ± SEM of the percentage of cells exhibiting SCs in the three groups; *, p< 0.05 (chi-Square test); c) mean values ± SEM of the number of SCs per cell in CTRL (*n*=42), NPc_5_ (*n*=47), and NPc_50_ (*n*=50); *, p< 0.05 vs CTRL (one-way ANOVA)

Also, based on the findings described above concerning V_r_ fluctuations in patched resting cardiomyocytes during 60 s time intervals, we investigated the possible occurrence of spontaneous contractions (SCs) in intact (not patched) cardiomyocytes. Figure 4a shows representative traces of sarcomere length in un-stimulated CTRL (top) and NPc_50_ (bottom) cells. Spontaneous activity in CTRL cells consisted of rare events (1 event during the 60 s time window on average), while both NPc groups often displayed many SCs (> 2 events / 60 s on average) of various amplitudes during the recording interval: the statistically significant twofold increase is reported in the histogram of panel c. Another way to look at the incidence of spontaneous mechanical activity is measuring the percentage of un-stimulated cells displaying SCs, which was significantly higher in NPc_5_ and NPc_50_ as compared to controls (Fig. 4b). Only occasionally, SCs exhibited an amplitude similar to the electrically induced contraction (see arrow in lower panel of Fig. 4a).

### ROS production induced by exposure to Co_3_O_4_-NPs

**Fig. 5:**
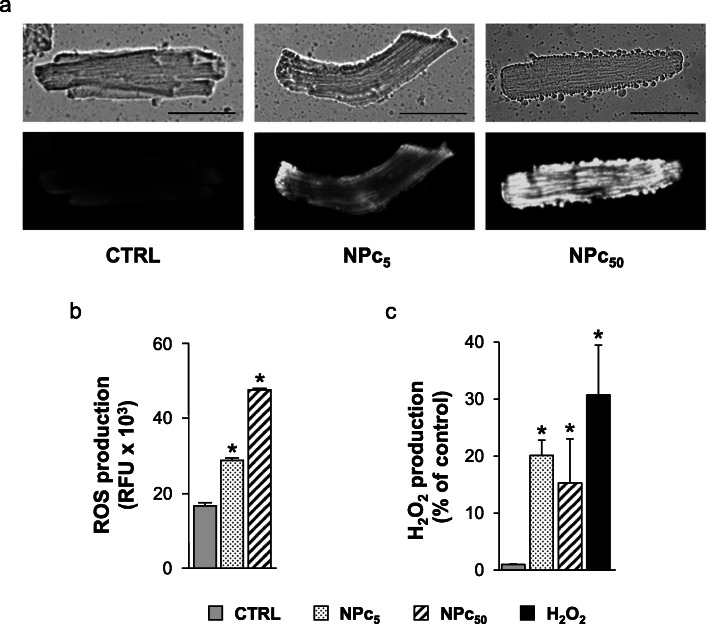
ROS production in rat ventricular cardiomyocytes exposed to Co_3_O_4_-NPs. Intracellular ROS amount was measured in untreated (CTRL) or NP-treated cardiomyocytes at 5 μg/ml (NPc_5_) and 50 μg/ml (NPc_50_) after 1 h exposure at room temperature using different methodologies. **a**) Cardiomyocytes were stained with CellROX® Orange Reagent and visualized with fluorescence microscopy. A representative phase contrast (upper-side) and fluorescence (lower-side) microscopy images were shown. Scale bars were set at 50 μm. **b**) Reactive species (ROS/RNS) production detected using DCFDA assay were expressed as relative fluorescence units (RFU). *, p< 0.05 vs CTRL (one-way ANOVA; Bonferroni post-hoc test). **c**) Amount of H_2_O_2_ produced by cardiomyocytes was detected using Amplex red Hydrogen Peroxide/Peroxidase assay and expressed as RFU increment (%) respect to untreated control. As positive control, cardiomyocytes treated for 20 min with 0.003% H_2_O_2_ (0.88 μM) were also analyzed. *, p< 0.05 vs CTRL (Kruskal-Wallis test followed by U-Mann Whitney test)

It is well established that oxidative stress may have a crucial role in NP-induced toxicology [32–34]. In the present work, we used different methodologies to evaluate the possibility that ROS could be induced in freshly isolated left ventricular cardiomyocytes treated for 1 h at room temperature with Co_3_O_4_-NPs (NPc_5_ e NPc_50_). Firstly, the activation of oxidative stress in living cells upon Co_3_O_4_-NP exposure was evaluated using the dye CellROX® Orange Reagent, which allows to detect several types of ROS in vivo. The increase in CellROX fluorescence associated with the treatment with Co_3_O_4_-NPs (Fig. 5a) indicated a significant increased production, compared to control, of the intracellular ROS levels depending on the NP concentrations.

A dose-dependent accumulation of intracellular reactive species (Fig. 5b) was also detected and quantified in NP-treated cardiomyocytes using 2′,7′-dichlorodihydrofluorescein diacetate (DCFDA) assay, a general dye for the detection of ROS and reactive nitrogen species (RNS) (Fig. 5b). An increased DCF fluorescence compared to untreated control cells following 1 h exposure to NPs at 5 and 50 μg/ml (+ 50% and + 160%, respectively; Fig. 5b) was coherent with CellROX-dependent ROS detection (Fig. 5a). These results suggest that Co_3_O_4_-NPs causes increased oxidative stress in cardiomyocytes.

Finally, we used Amplex Red Hydrogen Peroxide/Peroxidase assay to assess whether Co_3_O_4_-NPs specifically determined an increase in the production of hydrogen peroxide (H_2_O_2_) in cardiomyocytes. We observed a significant increase (+ 20%, Fig. 5c) in the amount of H_2_O_2_ synthesized by cardiomyocytes exposed to Co_3_O_4_-NPs, which reaches its maximum already at the lowest dose of NPs (NPc5; Fig. 5c).

### DNA damage induced by exposure to NPs

**Fig. 6:**
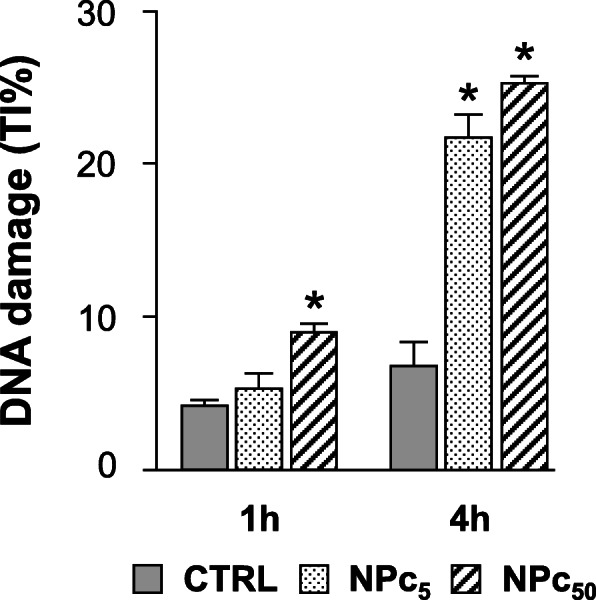
DNA damage induced by acute Co_3_O_4_-NP exposure of rat ventricular cardiomyocytes. DNA damage detected by Comet assay in untreated (CTRL) or Co_3_O_4_-NP treated cardiomyocytes at 5 μg/ml (NPc_5_) and 50 μg/ml (NPc_50_), after 1 h and 4 h exposure. DNA damage is expressed as tail intensity (TI%); *, p< 0.05 vs CTRL (one-way ANOVA; Bonferroni post-hoc test)

To evaluate if the treatment with Co_3_O_4_-NPs could induce DNA damage in freshly isolated cardiomyocytes after acute administration (1–4 h) of NPs, we used the alkaline Comet Assay (Fig. 6). Specifically, just after 1 h of exposure, we observed a significant increase in nuclear DNA damage in cardiomyocytes treated at highest dose (NPc_50;_ + 100%). Data collected after 4 h treatment showed a significant increase in DNA damage in both NPc_5_ and NPc_50_ cardiomyocytes (+ 214% and + 257% respectively; Fig. 6).

### Intracellular NP detection

**Fig. 7:**
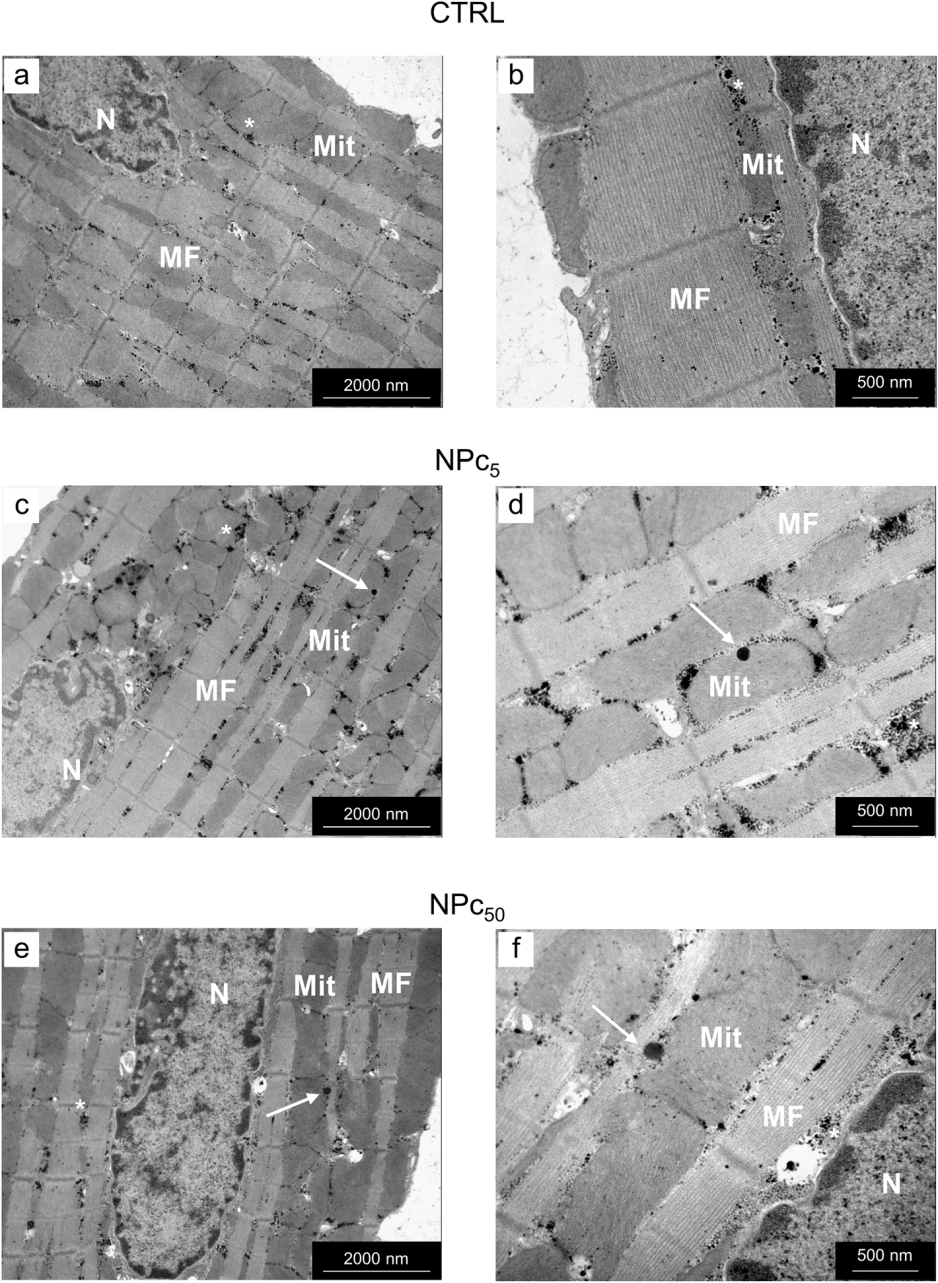
Ultrastructural detection of Co_3_O_4_-NPs in rat ventricular cardiomyocytes. Panels **a** and **b**: Transmission Electron Microscopic (TEM) images of a CTRL cardiomyocyte illustrating the regular distribution of mitochondria (Mit) and myofibrils (MF) and the scattered accumulation of small electrondense glycogen granules (*). Panels c-f: Co_3_O_4_-NPs (arrows) were detected within the mitochondria (Mit; **c**-**d**) and at the interface between mitochondria and myofibrils (MF; e-f) in cardiomyocytes exposed to 5 μg/ml (NPc_5_) and 50 μg/ml (NPc_50_) Co_3_O_4_-NPs, respectively. N: cardiomyocyte nucleus. Scale bars: 2 μm in a, c, d; 0.5 μm in **b**, **d**, **f**.

The ultrastructural analysis documented the presence of Co_3_O_4_-NPs within isolated NPc_5_ and NPc_50_ cardiomyocytes (Fig. 7). Internalized electrondense NPs, 15–20 nm in diameter, were found within inter-myofibrillar mitochondria (IFM, see arrows in panels c and d) and at the boundary between mitochondria and myofibrils (see arrows in panels e and f), where Intracellular Energetic Units (ICEU) and calcium microdomains are known to be located. This finding suggests that Co_3_O_4_-NPs might negatively affect cardiomyocyte function by a direct interaction with fundamental subcellular structures.

## Discussion

Our findings indicate that Co_3_O_4_-NPs can: i) enter cardiomyocytes within 1 h of exposure, ii) induce ROS production and DNA damage, and iii) alter cellular electrophysiological and mechanical properties.

An implicit assumption of our study is that cobalt NPs do reach the cardiac compartment when inhaled. Previous studies support this assumption in the case of different NP types [47, 49, 50]; we refer to the TEM data on cardiac tissue of a Co_3_O_4_-NPs inhaled rat (supplemental Fig. S1) and control (supplemental Fig. S2) as a further supporting proof (see also corresponding supplemental legends). Note the presence of NPs within myofibrils (panels e and f of Fig. S1), at the boundary between myofibrils and inter-fibrillar mitochondria (panels a and b), and within mitochondria (panels c and d).

Currently, Co_3_O_4_-NPs are increasingly used in several technological applications, due to their peculiar properties [1, 10–16]. It is now well known that many types of NPs can pass the alveolar barrier depending on their size and, through the blood flow, reach several body districts including the heart [47, 49]. It has also been shown that Co_3_O_4_-NPs can cross the plasma membrane by endocytotic and non-endocytotic mechanisms [51]. Until now, Co_3_O_4_-NPs toxicity was mostly attributed to an enhanced ROS production and mitochondrial dysfunction, resulting in impaired ATP production [33, 34]. ROS, including H_2_O_2_, are critical signaling molecules with important roles in both cardiac physiology and disease. Under physiological conditions, ROS signaling regulates heart development and cardiomyocyte maturation, cardiac calcium handling, excitation contraction coupling, and vascular tone. However, in pathological conditions increased ROS levels can result in oxidative stress through oxidative damage to DNA, proteins, and lipids, as well as activation of mitochondrial dysfunction, and cell death. Indeed, oxidative stress have been implicated in several cardiac diseases. In the present work we have not evaluated the cellular antioxidant response nor the activation of the DNA damage repair system in NP-treated cardiomyocytes, but the detrimental electrophysiological effects observed in cardiomyocytes after treatment with NPs lead us to hypothesize a pathological mechanism mediated by the production of ROS.

Also, increased ROS levels have been shown to raise the open probability of Ryanodine Receptors (RyR) and, with that, the likelihood of occurrence of spontaneous sarcoplasmic reticulum (SR) releases. We propose therefore that cobalt NPs cause an increase in the occurrence of spontaneous SR releases through two different pathways. Along the first pathway, the negatively charged Co_3_O_4_-NPs (see Zeta potential, Methods section) generate life-compatible transient nanopores across the cell membrane [52]. It is conceivable that this local membrane disassembly results in a slow but constant leak of Ca^2+^ along its electrochemical gradient and, through the sarcoplasmic reticulum calcium ATPase (SERCA), to a substantial increase in SR calcium content, thus increasing calcium gradient across RyRs, which promotes spontaneous SR Ca^2+^ releases, a recognized source of triggered arrhythmias in the heart [53]. Along the second pathway, as explained above, Co_3_O_4_-NPs also increase spontaneous SR calcium releases via increased RyR openings secondary to ROS augmentation.

We note that a slow increase in intracellular calcium via NP-induced nanopores is not necessarily expected to affect diastolic Ca^2+^ level nor sarcomere length: the latter was in fact found unmodified (see above), likely due to the high efficiency of removal mechanisms such as SERCA pump and sodium-calcium exchanger (NCX), with contribution of mitochondrial uniporters [53].

An increase in spontaneous SR Ca^2+^ releases coherently explains: i) the significant increase in the number of SCs recorded in the absence of electrical stimulation in NPc_5_ and NPc_50_ cells, as well as the higher percentage of cobalt treated cardiomyocytes displaying SCs (Fig. 4), and ii) the increased V_r_ instability observed in resting treated ventricular myocytes.. SR calcium overload is in fact expected to increase local calcium release events, in the form of stochastic sparks all over the cell volume, causing sporadic fluctuations in sub-sarcolemmal calcium mainly via the electrogenic activity of the Sodium Calcium Exchanger [54], which we reveal in high frequency oscillations of V_r_ (Fig. 1). Also, when SR calcium content rises above a certain threshold, the calcium sparking activity tends to synchronize along SR membrane, causing spontaneous calcium releases comparable to those normally elicited during EC coupling, as we actually observed in NP-exposed cells in the absence of electrical stimulation (Fig. 4).

Concerning the significant decrease in C_m_ that we found in NPc_50_ cells (Fig. 2b), we have hypothesized in a previous study [49] that nanopores production is likely accompanied by NP-internalization involving a net loss of membrane.

Furthermore, the remarkable dose-dependent shortening in APD in both the early (APD_-20mV_) and late phase (APD_-60mV_) of repolarization observed in Co_3_O_4_-NPs treated cells can be explained by the decrease in the depolarizing L-type Ca^2+^ current due to enhanced Ca^2+^-dependent inactivation, secondary to the greater amount of calcium released from the SR.

As for the dose-dependent impaired cell contractility, associated with an increased calcium transient and a prolonged τ observed in Co_3_O_4_-NP treated cardiomyocytes, several factors should be taken into consideration to explain this finding. First of all, the physical interaction between NPs and the myofilaments. In fact, as demonstrated by TEM images, Co_3_O_4_-NPs penetrated the sarcolemma and reached the cytosol by establishing intimate contact with mitochondria (note the interaction with inter-myofibrillar mitochondria, mainly involved in the production of ATP for cardiomyocyte contraction, in Fig. 6) and myofibrils, potentially interfering with the contractile proteins function. More importantly, Co_3_O_4_-NPs were found at the boundary between mitochondria and myofibrils, where Intracellular Energetic Units (ICEU) and calcium microdomains are known to be located and whose function is deeply intertwined with the cycle of SR calcium fluxes. Secondly, Co_3_O_4_-NPs determined cardiomyocyte DNA damage and increased ROS levels, leading to mitochondrial dysfunction and reduced ATP production. ATP must be generated in large amount to support the viability and contractile function of the myocardium. ATP, mostly derived from β-oxidation of free fatty acids and oxidative phosphorylation in the mitochondria [55], is consumed in the sarcomere contractile process (60–70%), as well as for the function of various ion pumps such as SERCA (30–40%) [55]. A decreased ATP availability determines a lower SERCA activity with a consequent prolongation of the time required for cytosolic Ca^2+^ removal, as we observed (τ values). Increased ROS levels act also in this case synergistically with lower ATP levels in downregulating SR calcium uptake [56].

Finally, we note that the reduced ATP availability, together with the interference between Co_3_O_4_-NPs and myofilaments discussed above, explain why the observed increase in the amplitude of calcium transients in NP exposed cells was not paralleled by increase in cell shortening which, on the contrary, was found decreased (panels b and g of Fig. 3).

The similarity in the functional outcomes of cobalt NP exposure respect to our previous report on titanium NPs, that share size, Zeta potential and solubility, and particularly the low dissolution of these NPs (< 1%, Methods section), suggest the intriguing possibility that harmful effects at the cellular level can be mediated chiefly by their physical rather than chemical properties. It is also along this line that future research on environmental toxicology should be devoted.

## Conclusions

Given the growing attention gained by the properties, and industrial/bio-medical applications [1–3] of NPs, an in-depth knowledge of their action at the cellular level is needed in order to better define NP toxicity [4–7] and support the growth of sustainable and safe nanotechnologies, limiting the health risk associated with daily exposure of subjects/workers. Along this line, the present study draws light on the interaction between Co_3_O_4_-NPs and cardiac tissue. Consistently with our previous work on cardiac effects of titanium dioxide (TiO_2_) NPs [49], we show here that Co_3_O_4_-NPs can directly and indirectly, via cellular oxidative stress, affect cardiomyocyte functional properties, leading to alterations in intracellular calcium handling, and reduced electromechanical efficiency, with significance for propensity to arrhythmias. Further studies are required to better evaluate mitochondrial involvement, as well as the effects of long-term exposures.

## Study limitations

Although we achieved the primary goal of the study, i.e. showing the negative direct effect of Co_3_O_4_-NPs on electromechanical properties of isolated ventricular myocytes, we acknowledge that some of the proposed mechanisms are merely speculative. For example, we speculated that the mitochondrial dysfunction and the consequent reduced ATP cellular levels contribute to the dose-dependent depression of cell contractility. We note however that our hypothesis is supported by the finding that treated cardiomyocytes exhibited high level of ROS suggesting an induction of oxidative stress, which is well known to determine mitochondrial dysfunction and reduce ATP availability in different heart diseases. Also, our hypothesis of calcium leaking into the cell is speculative but it does explain the observed changes in excitation-contraction coupling after NP-exposure, and is consistent with the lack of changes in resting membrane potential. Even though we cannot exclude the role of other ions leaking through nanopores, only calcium is rapidly and largely electro-neutrally removed from the cytoplasm (by SERCA). The restoration of any other ion gradient would in fact require electrogenic mechanisms, such as NaK pump, which would in turn modify the average value of resting membrane potential that we did not observe.

## Methods

This study was carried out in accordance with the recommendations of the Guide for the Care and Use of Laboratory Animals of the National Institute of Health. The protocol was approved by the Veterinary Animal Care and Use Committee of the University of Parma and conforms to the National Ethical Guidelines of the Italian Ministry of Health (Permit: n. PMS 53/2009). All effort was made to minimize suffering.

### Experimental animals

Experiments were performed on 32 male Wistar rats bred in our departmental animal facility, aged 12–14 weeks and weighing 300–350 g. The animals were kept in single-sex groups of four individuals from weaning (4 weeks after birth) until the onset of the experiments, in a temperature-controlled room at 20–24 °C, with the light on between 7.00 AM and 7.00 PM. The bedding of the cages consisted of wood shavings; food and water were freely available. Rats were anesthetized with a mixture of 40 mg/kg ip ketamine chloride (Imalgene, Merial, Milano, Italy) and 0.15 mg/kg ip medetomidine hydrochloride (Domitor, Pfizer Italia S.r.l., Latina, Italy) and, after decapitation, the hearts were excised and LV myocytes were enzymatically isolated, incubated with two different concentrations of Co_3_O_4_-NPs (5 μg/ml and 50 μg/ml – see below), and used for measuring cellular electrophysiological properties (20 rats) and cardiomyocyte contractility and calcium transients (12 rats). Fractions of isolated cardiomyocytes were also utilized for ROS quantification, genotoxicity (COMET assay), and the presence of Co_3_O_4_-NPs in single cardiomyocytes (transmission electron microscopy-TEM). Two rats were also instilled with saline and 2 mg/Kg Co_3_O_4_-NP solutions respectively and used for TEM analysis of left ventricular tissue. For the instillation procedure and tissue TEM protocol, see reference 49.

### Particle suspension

Co_3_O_4_-NP powder was suspended in sterilized high**-**purity water at a concentration of 2.5 mg/ml. Immediately before each experiment the NP suspension was vortexed, kept in a vial immersed on ice, and directly sonicated (Branson Ultrasonics,3 Danbury, CT, USA) through five 20 s lasting cycles at 65% of the maximum effective power (70 W), in order to minimize particle agglomeration and avoid temperature increase.

### Characterization of Co_3_O_4_-NPs

Co_3_O_4_-NPs (Cobalt (II,III) oxide, code: 637025, Sigma-Aldrich, Milan, Italy) used in the present work were previously characterized [57, 58]. Briefly, TEM analysis showed that Co_3_O_4_-NPs exhibited an irregular (non-spherical) shape with a mean diameter of 17 nm and the formation of small agglomerates of tens of NPs. The estimated specific NP surface areas was 46.7 m^2^/g. Zeta potentials and particle size distribution of the NP dispersions were determined by dynamic light scattering (DLS) technique using a NanoBrook 90Plus PALS analyzer (Brookhaven Instruments Corporation, Holtsville, NY, USA). Co_3_O_4_-NPs showed an average hydrodynamic diameter of 233 nm and a zeta potential of − 19 mV in aqueous medium suggesting that Co_3_O_4_-NP suspension was moderately stable and that these NPs had a negative charge.

Co_3_O_4_-NP solutions (concentration range: 10–100 μg/ml) prepared in aqueous medium were incubated for 24 h with gently shaking. After centrifugation (10′ at 21000 *g*), supernatants were collected and Co^2+^ amount determined using an inductively coupled plasma/mass spectrometry (ICP-MS) analysis (ELAN DRC II, Perkin Elmer SCIEX, Shelton, CT, USA) as indicated in [58]. Very low amounts of Co^2+^ ions (390 ng/ml; 0.78% of the total amount) were found in the centrifuged supernatants of these NP preparations indicating low rate of dissolution.

### Cardiomyocyte isolation and treatment

Single left ventricular myocytes were enzymatically isolated by collagenase perfusion, in accordance with a procedure previously described [59]. Briefly, the heart was rapidly excised and perfused at 37 °C by means of an aortic cannula with the following sequence of solutions gassed with 100% oxygen: 1) a calcium-free solution for 5 min to remove the blood, 2) low-calcium solution (0.1 mM) plus 1 mg/ml type 2 collagenase (Worthington Biochemical Corporation, Lakewood, NJ, USA) and 0.1 mg/ml type XIV protease (Sigma-Aldrich) for about 20 min, and 3) an enzyme-free, low-calcium solution for 5 min. Calcium-free solution contained the following (in mM; all chemicals, where not differently stated, were purchased from Sigma-Aldrich): 126 NaCl, 22 dextrose, 5.0 MgCl_2_, 4.4 KCl, 20 taurine, 5 creatine, 5 Na pyruvate, 1 NaH_2_PO_4_, and 24 HEPES (pH = 7.4, adjusted with NaOH). The left ventricle was then minced and shaken for 10 min. Cells were filtered through a nylon mesh and re-suspended in low**-**calcium solutions for 30 min (0.1 and 0.5 mM, for 15 min each). Proper aliquots (in the range of μl) of sonicated stock solution containing Co_3_O_4_-NPs were added with a micropipette right below the surface of the cardiomyocyte suspension contained in 50 ml tubes, in order to reach the desired final concentrations (5 μg/ml and 50 μg/ml). Cardiomyocytes were suspended immediately after the addition, and then maintained in gentle shaking for the entire incubation time (at least 1 h) in order to achieve uniform spatial mixing of Co_3_O_4_-NPs, maximize contact surface, and allow NP internalization, which was eventually verified by TEM analysis (see below). The same procedure was applied to CTRL cells. Since data on Co_3_O_4_-NPs bioaccessibility and bioavailability in vivo are still lacking, NP doses used in the present work were chosen according to the literature [33, 34, 60–63]. The lower dose (5 μg/ml, NPc_5_) is known to determine cytotoxic and genotoxic effects and inflammatory response on different cultured cell types while the higher (50 μg/ml, NPc_50_) was chosen in order to test dose-dependent effects. The concentration range adopted in our study is anyway in line with or lower than those used in previous in vitro works, in which the effects of Co_3_O_4_-NPs were investigated on different cell types including not only human cancer cells, endothelial cells derived from human aorta and umbilical vein, and mouse fibroblasts, but, most importantly, circulating cells like angiogenic cells and peripheral blood neutrophils [33, 34, 60–63]. Cells were then placed in a chamber mounted on the stage of an inverted microscope (Nikon-Eclipse TE2000-U, Nikon Instruments, Florence, Italy) and superfused (1 ml/min at 37 °C) with a Tyrode solution containing (in mM): 140 NaCl, 5.4 KCl, 1 MgCl_2_, 5 HEPES, 5.5 glucose, and 1.8 CaCl_2_ (pH = 7.4, adjusted with NaOH). Mechanical and electrical properties were then determined in control (CTRL) and treated cells at 5 μg/ml (NPc_5_) and 50 μg/ml (NPc_50_). All experiments were performed within 6 h after isolation. All cardiomyocytes used were rod-shaped and showed well**-**defined striations.

### Patch-clamp technique

CTRL and NP_C_, perfused as described above, were brought in whole-cell patch-clamp configuration and transmembrane potential (V_m_) was measured in current-clamp mode using a Multiclamp 700B amplifier (Axon Instruments, Union City, CA, USA). Suction pipettes were made from borosilicate capillary tubing (Harvard Apparatus LTD, Edenbridge, UK) with an access resistance of 2 to 4 MΩ when filled with a solution containing the following (in mM): 113 KCl, 10 NaCl, 5.5 dextrose, 5 K_2_ATP, 0.5 MgCl_2_, and 10 HEPES (pH = 7.1 adjusted with KOH). Data recordings and analysis were performed via Clampfit9 software (Molecular Devices, Sunnyvale, CA, USA). Stability of V_r_ was assessed by measuring percent deviation of V_m_ data, sampled for 20 s intervals at 10 kHz in un-stimulated patched cells, from the normal distribution, which is expected in normal resting conditions. Cells were previously conditionally paced at 5 Hz for 8 s, in order to establish a common sarcoplasmic calcium filling for better comparison. Membrane capacitance (C_m_) was derived according to a protocol previously described [64] by means of 100 ms-long hyperpolarizing constant current pulses. Sequences of action potentials (APs) were elicited by injection of brief (3 ms) depolarizing current pulses of amplitude 1.5 times the current threshold and sampled at 10 kHz. The pacing rate of 5 Hz (cycle length CL = 200 ms) is close to physiological heart rate in rat.

Time was allowed for AP waveforms to reach a steady state configuration (usually within 30 beats). Then, 10 consecutive APs were recorded and averaged, and the following parameters measured: i) resting membrane potential (V_r_, mV), ii) action potential amplitude (APA, mV) as the difference between AP upstroke and the preceding V_r_, and iii) AP duration at − 20 mV (APD_-20mV_) and − 60 mV (APD_-60mV_), representative of the early and late phase of repolarization respectively. APD was calculated as the interval between the time of maximal upstroke velocity (dV/dt_max_) and the time when membrane potential (V_m_) reached − 20 and − 60 mV during repolarization. Beat-to-beat variability of APD_-60mV_, was calculated as the coefficient of variability of APD_-60mV_ of the10 consecutive APs (CV_-60mV_) [65, 66].

### Cardiomyocyte contractility and Ca^2+^ transients

Cell mechanical properties were evaluated via the IonOptix fluorescence and contractility system (IonOptix, Milton, MA, USA). Cells were field-stimulated at 0.5 and 1 Hz pacing frequency by constant depolarizing pulses (2 ms in duration, and twice diastolic threshold in intensity) delivered by platinum electrodes placed on opposite sides of the chamber connected to a MyoPacer Field Stimulator (IonOptix). The stimulated myocyte was displayed on a computer monitor using the IonOptix MyoCam camera. The shortening of unloaded cardiomyocytes was measured with the IonOptix system, which captures sarcomere length dynamics via a Fast Fourier Transform algorithm. Sampling rate was fixed at 1 KHz. A total of 232 isolated ventricular myocytes were analyzed (73 CTRL, 71 NP_C5_ and 88 NP_C50_) to compute the following parameters: mean diastolic sarcomere length, fraction of shortening (FS, %), maximal rates of shortening (−dL/dt_max_, μm/s), and re-lengthening (+dL/dt_max_, μm/s), time of total cycle measured at 90% of re-lengthening (T-cycle_90%_, s) and re-lengthening time measured from the peak of contraction (T-rel_90%_, s).

In a subset of cells of each group (40 CTRL, 35 NP_C5_, and 21 NP_C50_), Ca^2+^ transients were measured simultaneously with cell motion, by means of epifluorescence recordings. Ca^2+^ epifluorescence was measured after loading the myocytes with fluo-3-AM (10 μΜ; Invitrogen, Carlsbard, CA, USA) for 30 min. Excitation length was 480 nm, with emission collected at 535 nm using a 40x oil objective lens (NA 1:3). Fluo-3 signals were expressed as normalized to baseline fluorescence emitted at rest (f/f0: fold increase). The time course of the fluorescence signal decay was described by a single exponential equation, whose time constant (τ) measures the rate of intracellular Ca^2+^ clearing [67].

The occurrence of spontaneous contractions was evaluated for 60 s in the absence of electrical stimulation after a 60 s conditioning pacing at 2 Hz in 42 CTRL, 47 NP_C5_, and 50 NP_C50_.

### ROS quantification

Freshly isolated left ventricular cardiomyocytes were treated (or not) for 1 h at room temperature with Co_3_O_4_-NPs (NPc_5_ e NPc_50_) and ROS species were measured using general oxidative stress indicators (CellROX® Orange Reagent; DCFDA) and Amplex Red Hydrogen Peroxide/Peroxidase assay for the quantification of H_2_O_2_ synthesized by cardiomyocytes.

#### CellROX™ assay

Cardiomyocytes treated (or not) with Co_3_O_4_-NPs were stained for 30 min at 37 °C with CellROX™ Orange Reagent (5 μM, final concentration) according to manufacturer’s instructions (Thermo Fisher Scientific, CA, USA). This cell-permeant dye is non-fluorescent in a reduced state and exhibits bright orange fluorescence (with excitation/emission at 545/565 nm) upon oxidation mediated by ROS generated by various agents, including NPs [68]. Image acquisition and analysis were performed with a Zeiss Axio Imager.Z2 fluorescence microscope (Carl Zeiss Microscopy GmbH, Jena, Germany). To ensure reproducibility between experiments, all images were recorded with the same microscope settings.

#### DCFDA assay

DCFDA (Sigma-Aldrich, Merck KGaA, Darmstadt, Germany) is a cell-permeant non-fluorescent probe that is de-esterified intracellularly and then oxidized by ROS and RNS to its fluorescent form (with excitation/emission at 480/538 nm). ROS production was measured in cardiomyocytes treated (or not) with Co_3_O_4_-NPs using DCFDA (Sigma-Aldrich, Merck KGaA, Darmstadt, Germany) as described by Giordano et al. [69]. In a typical experiment, cardiomyocytes were washed with PBS and then pre-incubated for 30 min (37 °C) with DCFDA (20 μM), which was added from a stock solution in DMSO and diluted in PBS. The quantity of DMSO never exceeded 0.1% and was also added to the blank. Extracellular DCFDA was removed by washing the cells with HBSS (Sigma-Aldrich). After 1 h at 37 °C, incubation medium (HBSS) was removed and a solution of Tris-HCl-TritonX100 and a cell dissociation solution (Sigma-Aldrich) were added for 10 min. After centrifugation, the supernatant was collected, and fluorescence was immediately read with a fluorescence microplate reader (TECAN SpectraFluor Plus, Männedorf, Switzerland).

#### Amplex red hydrogen peroxide/peroxidase assay

H_2_O_2_ synthesized by cardiomyocytes and released into the supernatant was quantified using Amplex red Hydrogen Peroxide/Peroxidase assay according to manufacturer’s instructions (Thermo Fisher Scientific). Briefly, cardiomyocytes (1.5*10^4^) treated (or not) with Co_3_O_4_-NPs were resuspended in 20 μl Krebs–Ringer phosphate glucose buffer (KRPG; 145 mM NaCl, 5.7 mM sodium phosphate, 4.86 mM KCl, 0.54 mM CaCl_2_, 1.22 mM MgSO_4_, 5.5 mM glucose, pH 7.4) and added to 100 μl of reaction mixture (containing 50 μM Amplex® Red reagent and 0.1 U/mL horseradish peroxidase in KRPG), prewarmed at 37 °C for 10 min. Samples were incubated at 37 °C for 1 h and the fluorescence was measured using a fluorescence microplate reader (TECAN SpectraFluor Plus). In the presence of peroxidase, the Amplex Red reagent reacts with H_2_O_2_ and produces resorufin, a red-fluorescent oxidation product (with excitation/emission at 535/595 nm). As positive control, cardiomyocytes treated for 20 min with 0.003% H_2_O_2_ (0.88 μM) were also analyzed with Amplex Red Hydrogen Peroxide/Peroxidase assay.

### Comet assay

DNA damage was measured using single-cell gel electrophoresis (SCGE, Comet assay). The alkaline version (pH > 13) of the assay was performed to detect single-strand breaks and alkali-labile sites, such as apyrimidinic and apurinic sites that are formed when bases are lost and oxidized. SCGE was performed basically according to Singh NP et al. [70], with some minor modifications applied to adapt the procedure to cardiomyocytes. Immediately after enzymatic isolation, cardiomyocytes were treated with Co_3_O_4_-NPs (5 μg/ml and 50 μg/ml). After incubation, cells were centrifuged (5 min, 800 g). Cell pellets were then resuspended in 1 ml of HBSS diluted 1:9 and incubated at room temperature (RT) for 30 min in order to prepare cardiomyocytes for the lysis before the last centrifugation (1 min, 800 g), which was needed to obtain a reasonable amount of cell pellets. The pellets were resuspended in 90 ml Low Melting Agarose 0.7% (LMA) and transferred onto degreased microscope slides previously dipped in 1% Normal Melting Agarose (NMA) for the first layer. The agarose was allowed to set for 15 min at 4 °C before addition of a final layer of low melting agarose (LMA). Cell lysis was carried out at 4 °C overnight by exposing cells to a buffer containing 2.5 mM NaCl, 100 mM Na_2_EDTA, 8 mM Tris-HCl, 1% Triton X-100 and 10% DMSO, pH=10. The electrophoretic migration was performed (DNA unwinding: 20 min; electrophoresis: 20 min, 0.78 Vcm^− 1^, 300 mA) in alkaline buffer, pH> 13, (1 mM Na_2_EDTA, 300 mM NaOH, 0 °C).

DNA was stained with ethidium bromide (10 μg/ml) before the examination at 400X magnification under a Leica DMLS fluorescence microscope (excitation filter BP 515–560 nm, barrier filter LP 580 nm), using an automatic image analysis system (Comet Assay III– Perceptive Instruments Ltd., Bury St Edmunds, UK).

Total percentage of fluorescence in DNA fragmentation tail (TI, tail intensity, %) provided representative data on the genotoxic effects. For each sample, coded and evaluated blind, 100 cells were analyzed. All steps of the comet assay were conducted under yellow light.

### NP detection in single cardiomyocytes by TEM

Freshly isolated cardiomyocytes were washed twice in low-calcium solution (0.1 mM), fixed in Karnovsky solution (4% formaldehyde, 5% glutaraldehyde), and treated as previously described [71]. High-power micrographs were utilized to detect the presence of Co_3_O_4_-NPs within the cardiomyocytes.

### Statistical analysis

The SPSS statistical package was used (International Business Machines Corporation, Armonk, NY, USA, version 25). Normal distribution of variables was checked by means of the Kolmogorov**-**Smirnov test. Data are reported as mean values ± standard error of the mean (SEM). Comparison among groups involved GLM ANOVA for repeated measurements (cellular electrophysiological properties, cell mechanics, and calcium transients), Kruskal-Wallis non-parametric statistical test followed by U-Mann Whitney test (variability of resting membrane potentials, ROS detection using Amplex red Hydrogen Peroxide/Peroxidase assay), chi-Square test (percentage of cells displaying spontaneous cell contractions), and one-way ANOVA followed by Bonferroni or Games-Howell (as appropriate) post-hoc individual comparisons (genotoxicity evaluation, ROS detection using DCFDA assay, and number of spontaneous contractions). A *p*-value < 0.05 was considered statistically significant.

## Supplementary Information

**Additional file 1: Supplementary Fig. S1.** TEM analysis of left ventricular tissue from cobalt NP inhaled rat. Panel a: low magnification image of a cardiomyocyte (N, nucleus) filled of mitochondria (Mit) and myofibrils (MF). On the left, collagen bundles (Col) are present in the interstitial space. Aggregates of Co_3_O_4_-NPs (asterisk) within myofibrils and mitochondria are shown at higher magnification in (b). Panel c: low magnification image of LV myocardium showing the sarcolemma (arrow) lining the surface of a cardiomyocyte with abundant mitochondria (Mit) and myofibrils (MF). Collagen bundles (Col) are present in the interstitial space where an endothelial cell (Ec) is lining a capillary lumen (RB, red blood cells). The black rectangle inscribes an area shown at higher magnification in (d) in which the asterisks indicate NPs located in the mitochondria (Mit). Panel e: image showing a detail of mitochondria (Mit) and myofribrils (MF) in an NP-treated cardiomyocyte. The black rectangle inscribes an area shown at higher magnification in (f) in which NPs are located within the myofibrils (asterisk). Scale Bars: c: 5 μm; a,e: 2 μm; d, f: 1 μm, b: 500 nm

**Additional file 2: Supplementary Fig. S2.** TEM analysis of left ventricular tissue from control rat. Panel a: low magnification image of a cardiomyocyte (N, nucleus) filled of mitochondria (Mit) and myofibrils (MF). In the interstitial space an endothelial cell (Ec) is lining a capillary lumen (RB, red blood cells). Panel b: image showing mitochondria (Mit) and myofribrils (MF) in a CTRL rat myocardium (N, myocyte nucleus) at higher magnification. Scale Bars: a: 5 μm; b: 2 μm.

## Data Availability

All relevant data are included in the manuscript. They are also available from the authors on reasonable request.

## Abbreviations

NPs: nanoparticles
Co_3_O_4_: cobalt oxide
Co_3_O_4_-NPs: cobalt oxide nanoparticles
ROS: reactive oxygen species
LV: left ventricular
NIOSH: National Institute of Occupational Safety and Health
ACGIH: American Conference of Governmental Industrial Hygienists
TEM: transmission electron microscopy
V_r_: resting membrane potential
CTRL: control cardiomyocytes
NPc_5_: Co_3_O_4_-NPs treated cardiomyocytes at 5 μg/ml
NPc_50_: Co_3_O_4_-NPs treated cardiomyocytes at 50 μg/ml
V_m_: transmembrane potential
AP: action potential
SD: standard deviation
C_m_: membrane capacitance
APA: action potential amplitude
APD: action potential duration
APD_-20mV_: action potential duration at − 20 mV
APD_-60mV_: action potential duration at − 60 mV
CV_-60mV_: coefficient of variability of APD_-60mV_
FS: fraction of shortening
-dL/dt_max_: maximal rate of shortening
+dL/dt_max_: maximal rate of re-lengthening
T-cycle_90%_: time of total cycle measured at 90% of re-lengthening
T-rel_90%_: re-lengthening time measured from the peak of contraction
f/f0: fold increase
τ: time constant
SCs: spontaneous contractions
RNS: reactive nitrogen species
DCFDA: 2′,7′-dichlorodihydrofluorescein diacetate
H_2_O_2_: hydrogen peroxide
IFM: inter-myofibrillar mitochondria
ICEU: intracellular energetic units
RyR: ryanodine receptor
SR: sarcoplasmic reticulum
SERCA: sarcoplasmic reticulum calcium ATPase
NCX: sodium-calcium exchanger
TiO_2_: titanium dioxide
IP: intra-peritoneal
DLS: dynamic light scattering
CL: cycle length
dV/dt_max_: maximal upstroke velocity
DMSO: dimethyl sulfoxide
HBSS: Hank’s Balanced Salt Solution
SCGE: single-cell gel electrophoresis
RT: room temperature
LMA: low melting agarose
NMA: normal melting agarose
EDTA: ethylenediaminetetraacetic acid
TI: tail intensity
SEM: standard error of the mean
