# Supplementary figures and images for: Cobalt oxide nanoparticles induce oxidative stress and alter electromechanical function in rat ventricular myocytes
Source: Part Fibre Toxicol. 2021 Jan 6;18:1. doi: 10.1186/s12989-020-00396-6 (PMC7788732; doi:10.1186/s12989-020-00396-6)

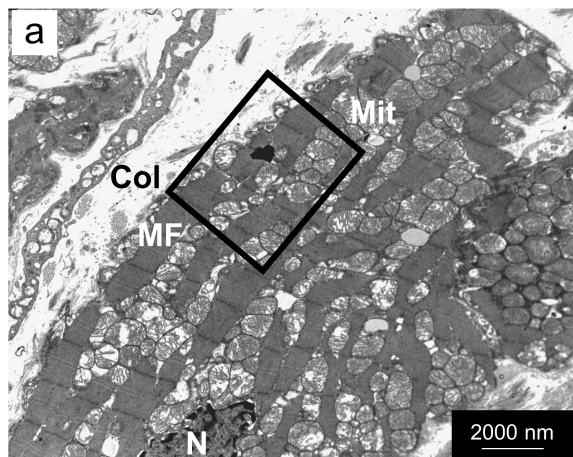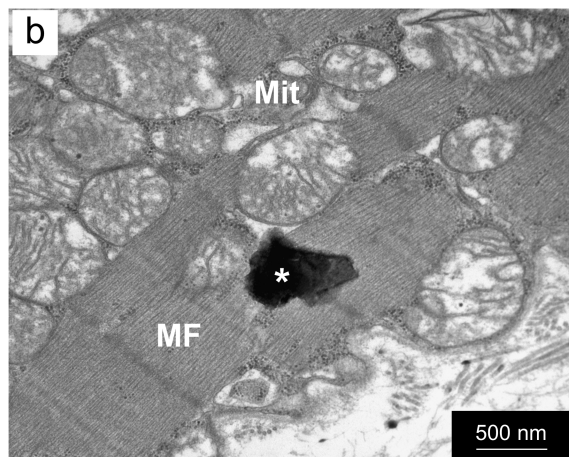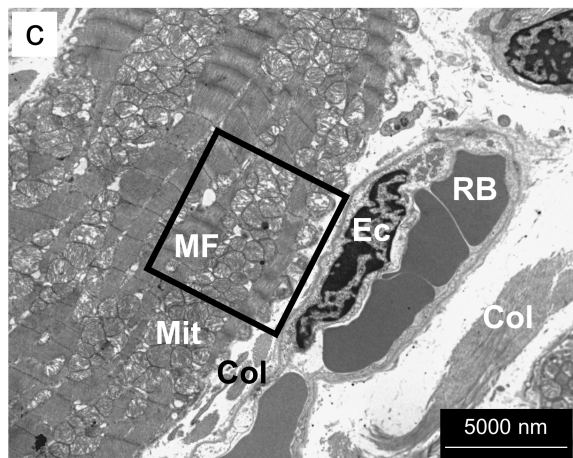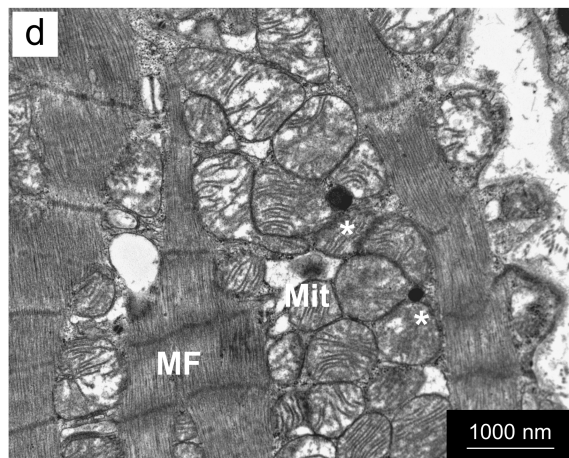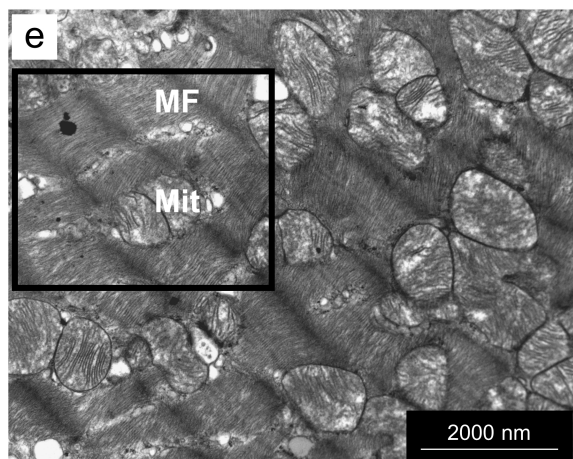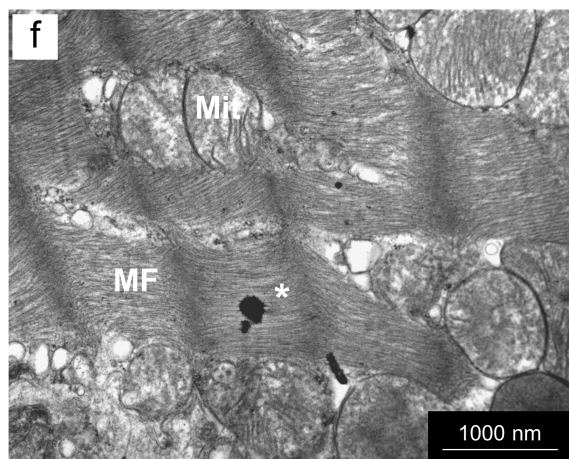

Supplement: Supplementary file 1 — Additional file 1: Supplementary Fig. S1. TEM analysis of left ventricular tissue from cobalt NP inhaled rat. Panel a: low magnification image of a cardiomyocyte (N, nucleus) filled of mitochondria (Mit) and myofibrils (MF). On the left, collagen bundles (Col) are present in the interstitial space. Aggregates of Co3O4-NPs (asterisk) within myofibrils and mitochondria are shown at higher magnification in (b). Panel c: low magnification image of LV myocardium showing the sarcolemma (arrow) lining the surface of a cardiomyocyte with abundant mitochondria (Mit) and myofibrils (MF). Collagen bundles (Col) are present in the interstitial space where an endothelial cell (Ec) is lining a capillary lumen (RB, red blood cells). The black rectangle inscribes an area shown at higher magnification in (d) in which the asterisks indicate NPs located in the mitochondria (Mit). Panel e: image showing a detail of mitochondria (Mit) and myofribrils (MF) in an NP-treated cardiomyocyte. The black rectangle inscribes an area shown at higher magnification in (f) in which NPs are located within the myofibrils (asterisk). Scale Bars: c: 5 μm; a,e: 2 μm; d, f: 1 μm, b: 500 nm [file 12989_2020_396_MOESM1_ESM.pdf]

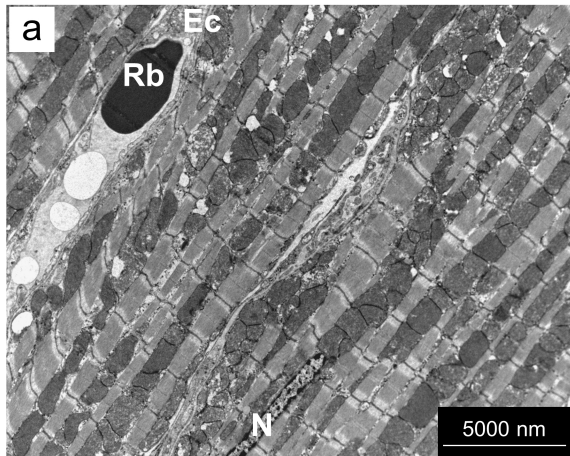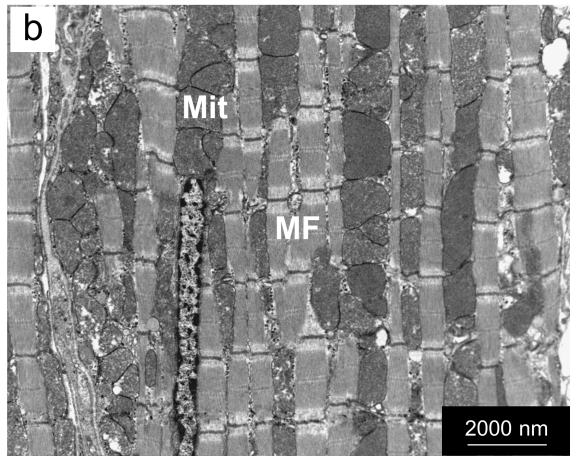

Supplement: Supplementary file 2 — Additional file 2: Supplementary Fig. S2. TEM analysis of left ventricular tissue from control rat. Panel a: low magnification image of a cardiomyocyte (N, nucleus) filled of mitochondria (Mit) and myofibrils (MF). In the interstitial space an endothelial cell (Ec) is lining a capillary lumen (RB, red blood cells). Panel b: image showing mitochondria (Mit) and myofribrils (MF) in a CTRL rat myocardium (N, myocyte nucleus) at higher magnification. Scale Bars: a: 5 μm; b: 2 μm. [file 12989_2020_396_MOESM2_ESM.pdf]
